# An open-label, randomized, placebo-controlled study on the effectiveness of a novel probiotics administration protocol (ProbiotiCKD) in patients with mild renal insufficiency (stage 3a of CKD)

**DOI:** 10.1007/s00394-018-1785-z

**Published:** 2018-08-03

**Authors:** Mariadelina Simeoni, Maria Lucia Citraro, Annamaria Cerantonio, Francesca Deodato, Michele Provenzano, Paola Cianfrone, Maria Capria, Silvia Corrado, Emanuela Libri, Alessandro Comi, Arturo Pujia, Ludovico Abenavoli, Michele Andreucci, Massimo Cocchi, Tiziana Montalcini, Giorgio Fuiano

**Affiliations:** 1 Nephrology Unit, Department of Surgical and Medical Science, ‘Magna Graecia’ University Hospital, Viale Europa, Germaneto Area, 88100 Catanzaro, CZ Italy; 2 Clinical Nutrition Unit, ‘Magna Graecia’ University Hospital, 88100 Catanzaro, CZ Italy; 3 Digestive Physiopathology Unit, ‘Magna Graecia’ University Hospital, 88100 Catanzaro, CZ Italy; 4 “Paolo Sotgiu” Institute for Research in Quantitative and Quantum Psychiatry and Cardiology, LUdeS, Lugano, Switzerland

**Keywords:** Chronic kidney disease, Uremia, Gut microbiota, Dysbiosis, Probiotics

## Abstract

**Purpose:**

Gut dysbiosis has been described in advanced, but not in initial stages of CKD. Considering the relevant impact of gut dysbiosis on renal and cardiovascular risk, its diagnosis and treatment are clinically relevant.

**Methods:**

We designed, open-label, placebo-controlled intervention study (ProbiotiCKD) to evaluate gut microbiota metabolism in a cohort of KDIGO CKD patients (*n* = 28) at baseline and after a randomly assigned treatment with probiotics or placebo. Gut microbiota status was evaluated on:.

**Results:**

Basal mean fecal *Lactobacillales* and *Bifidobacteria* concentrations were abnormally low in both groups, while urinary indican and 3-MI levels were, indicating a mixed (fermentative and putrefactive) dysbiosis. After treatment, mean fecal *Lactobacillales* and *Bifidobacteria* concentrations were increased, only in the probiotics group (*p* < 0.001). Conversely, mean urinary indican and 3-MI levels only in the group treated with probiotics (*p* < 0.001). Compared to placebo group, significant improvements of C-reactive protein (*p* < 0.001), iron (*p* < 0.001), ferritin (*p* < 0.001), transferrin saturation (*p* < 0.001), β2-microglobulin (*p* < 0.001), serum iPTH and serum calcium were observed only in the probiotics group.

**Conclusions:**

ProbiotiCKD is the first intervention study demonstrating that an intestinal mixed dysbiosis is present even in early CKD stage and can be effectively corrected by the novel mode of administration of high-quality probiotics with improvement of inflammatory indices, iron status and iPTH stabilization.

## Introduction

The human intestinal microbiota is a vast pool of symbiotic microorganisms living in the human gut and it is involved in important metabolic, trophic and immunological functions in the host [1]. 95% of microbiota is composed of anaerobic bacteria and 5% of aerobic bacteria [2]. Bacteria concentrations vary along the gut with an exponential increase in the fecal direction [3]. As for recent reports, fecal concentrations of *Lactobacillales* and *Bifidobacteria* range 1 × 10^7^ to 1 × 10^10^ CFU/g [4] and 1 × 10^8^ to 1 × 10^10^ CFU/g [5], respectively.

Gut dysbiosis consists of a significant alteration in microbiota composition and it is prevalently characterized by a concentration of *Lactobacillales* and *Bifidobacteria* and a prevalence of aerobic bacteria. Gut dysbiosis is associated with both intestinal and extra-intestinal reflexes. Of note, intestinal dysbiosis promotes atherosclerosis and hypertension and is involved in the activation of several molecular pathways of cardiovascular risk worsening [6].

Chronic kidney disease (CKD), a relevant multifactorial [7–9] health problem associated with poor quality of life [10], high management costs and increased death risk. In several studies conducted on both animals and humans, intestinal dysbiosis has been found in or end-stage renal disease (ESRD). Microbiota disequilibrium in ESRD patients depends on uremic and non-uremic factors, the latter based on dietary and pharmacological approach to CKD patients [11]. In advanced CKD stage vegetables and fruit intake has to be restricted to prevent the risk of hyperkalemia and fluid overload. This fiber shortage amplifies the predisposing factors to dysbiosis, such as intestinal transit slowing, intestinal wall edema and metabolic acidosis increase [12, 13]. Furthermore, ESRD imposes an increase of oral drugs (iron and vitamin D analogs supplementation, potassium and phosphate-chelating agents, diuretics) intake inducing pro-inflammatory gastrointestinal overload [14, 15]. Consequently, intestinal flora metabolism is greatly modified in uremic patients with prevalence of proteolytic and/or saccharolytic fermentation process with increased production and reabsorption of intestinal bacterial metabolites, such as indican and 3-methyl-indole (3-MI) [16, 17].

3-methyl-indole and indican are currently used to diagnose either a and/or a fermentative intestinal dysbiosis, respectively [18–23]. Fermentative dysbiosis is due to non-absorbed sugar hydrolization by several bacteria strains in the ascendant colon and in the caecum. Acetic acid, water and carbonic anhydride are produced by fermentation and cause intestinal wall edema, diarrhea, odorless meteorism and functional alteration of gut-associated lymphoid tissue (GALT) and mucosa-associated lymphoid tissue (MALT) [24]. Putrefactive dysbiosis, as previously described, is triggered by an overgrowth of intestinal putrefactive microorganisms favored by fecal pH changes and an unbalanced dietary intake of fibers, proteins and fats. The main symptoms of putrefactive dysbiosis are constipation, smelly meteorism, digestive difficulties, impaired GALT and MALT functions [25].

Moreover, it has been widely reported that metabolites of altered gut microbiota are directly involved in mechanisms of cardiovascular disease and renal damage progression [26–29].

We present the results of ProbiotiCKD study. This investigation was addressed to characterize gut microbiota status and metabolism in a cohort of patients with 3a stage CKD. ProbiotiCKD clinical trial tested the impact of a novel optimized probiotics administration protocol on urinary indican and 3-MI levels, all fecal *Lactobacillales* and *Bifidobacteria* concentrations, and serum biochemistry lab parameters.

## Materials and methods

**Fig. 1:**
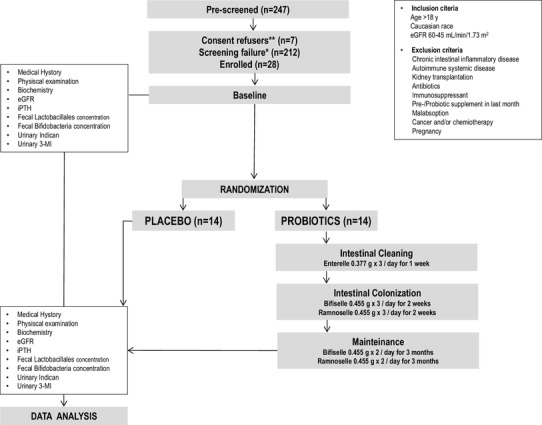
Study design *excluded after screening; **did not release the informed consent

We designed ProbiotiCKD protocol to be tested in a single-center, open-label, placebo-controlled intervention study. Patients with stable CKD stage 3a, referring to the Nephrology Unit at the University Hospital of Catanzaro (Italy), between January 2016 and March 2017, were progressively screened, as reported in Fig. 1. The eligible patients were studied to evaluate the impact on intestinal microbiota of a novel probiotics administration protocol vs placebo.

To be enrolled, patients had to meet the following criteria: age > 18 years; Caucasian race; Epidemiology Collaboration Equation (EPI) estimated-GFR (eGFR) ranging 60–45 ml/min/1.73 m^2^. Conversely, patients meeting Montreal classification criteria for inflammatory bowel diseases [30] were excluded. In addition, patients with malabsorption, autoimmune systemic diseases or cancer were excluded, as well as kidney transplant recipients, pregnant women and patients on current or recent antibiotic therapy or immunosuppressant drugs. To standardize baseline conditions, any prebiotic or probiotic supplement had to be suspended at least 1 month before enrollment. However, according to current nutritional recommendations to CKD patients [31, 32] and to obtain a satisfactory dietary prebiotic intake, the enrolled subjects had to adhere to a protein dietary intake ranging 0.7–1 g/kg/day, also assuring a daily consumption of two pieces of fruit (apple or pear) and 200 g of double-boiled leafy green vegetables (the double boiling was used to discharge vegetables’ potassium content) [33]. Patients were provided with a weekly dietary diary to be filled in. The diaries had to be analyzed by our nutritionist, to make sure that background dietary conditions were adequate and uniform.

Eligible patients agreeing protocol requirements, had to provide written informed consent to participate in the ProbiotiCKD study, which was conducted in accordance with the Helsinki Declaration.

At baseline, demographic data, medical history, dietary diaries and concomitant therapy were collected. A careful physical examination was also performed. In addition, a fasting peripheral venous blood sample was drawn for serum biochemistry and plasma intact parathormone (iPTH) assessment. Basal eGFR was calculated by CKD-EPI equation. In addition, a cultural quantitative analysis of two different fecal bacteria clusters (*Lactobacillales and Bifidobacteria*) were rapidly analyzed by anaerobic technique at the Microbiology Unit of University Hospital of Catanzaro. According to previous studies, any stool sample in which *Lactobacillales* and *Bifidobacteria* concentrations were found < 1 × 10^7^ CFU/g and < 1 × 10^8^ CFU/g respectively, was considered associated to an altered gut microbiota [4, 5, 22]. All patients had to collect a morning spot urine sample for 3-MI and indican assessment by high-performance liquid chromatography and colorimetric technique, respectively. As for the 3-MI determination, aliquots of 300 µl of acetonitrile were added to an equal volume of each urine sample. The obtained solutions were vortexed for 3 × 5 s and chilled at 4 °C for 15 min. Afterwards, samples were centrifuged for 5 min at 12,000 rpm (1 °C). Clear supernatants were then used to perform chromatography analysis on an Agilent 1100 LC-MS system. Chromatographic separation was obtained on a Waters XBridge column (3.5 µm, 4.6 × 150 mm; Waters, Milford, MA, USA), operating at 40 °C [34].

For urinary indican determination, a standard colorimetric assay kit was used according to the procedures indicated by the manufacturer (https://www.sigmaaldrich.com/content/dam/sigma-aldrich/docs/Sigma/Bulletin/2/mak128bul.pdf). According to our laboratory reference ranges and to previous observations [23, 35–39], urinary indican and 3-MI were considered normal for values lower than 10 and 10 µg/l, respectively. Creatininuria was also measured to calculate the urine indican and 3-MI to creatinine ratios.

Patients were randomized by a computer-generated random number list to receive either placebo or probiotics. Probiotics are defined by the United Nations Food and Agriculture Organization and the World Health Organization as “live microorganisms” that, when administered in adequate amounts, confer health benefits to the host [40, 41]. The use of different complexes of high concentration and stable probiotics in a ‘sequential’ manner was the novel administration mode tested in ProbiotiCKD study. The probiotics administration protocol was designed by nephrologists, nutritionists and gastroenterologists at our University Hospital, after an accurate review of the current literature.

In particular, the treated group underwent the following three phases of the treatment including the ‘sequential’ use of different probiotics with: (a) phase 1: intestinal cleaning by oral administration during main meals (breakfast, lunch and dinner) for 1 week of one capsule of a complex of probiotics (Enterelle 0.377 g ^®^Bromatech) composed of *Enterococcus faecium* (UBEF-41), *Lactobacillus acidophilus* (LA-14) and *Saccharomyces cerevisiae subspecies Boulardii* (MTCC-5375); (b) phase 2: intestinal colonization with oral administration, for 2 weeks, during breakfast, lunch and dinner of one capsule of a complex of *Bifidobacteria* (Bifiselle ®Bromatech 0.455 g) composed of *Bifidobacterium brevis* (BB03), *Bifidobacterium bifidum* (BB06), *Bifidobacterium longum* (BL05) and one capsule of another probiotics complex (Ramnoselle ^®^Bromatech 0.455 g) composed of *Lactobacillus rhamnosus* (HN-001), *Lactobacillus rhamnosus* (LR-32) and *Lactobacillus acidophilus* (LA-14); (c) phase 3: microbiota maintenance by both Bisifelle and Ramnoselle oral administration, one capsule of each, twice per day during breakfast and dinner for 3 months. Patients in the treated group were provided with plastic bottles containing the exact number of capsules necessary to complete the treatment. The choice of using *Lactobacillales* and *Bifidobacteria* was driven by scientific evidence. In fact, several studies have demonstrated a healthy-orientated intestinal environment modulation by using probiotic products containing *Bifidobacterium* and *Lactobacillus* strains [42].

Patients randomized to receive the placebo were provided with the same amount of placebo capsules covering and miming the entire treatment cycle with probiotics. Placebo capsules were prepared ad hoc by a galenic local pharmacy to look exactly as probiotic capsules. Even placebo plastic containers looked exactly as those of probiotics. To assure that patients had not discontinued capsules assumption during the treatment period, and to check if any side effect had occurred, the nutritionist (T.M.) in our team had to phone each patient daily during the first week of treatment, and every week afterward. At the end of treatment cycle, both groups underwent a follow-up visit in which a re-evaluation of all parameters assessed at baseline, was performed. Any capsule not taken by the patients had to be returned and counted at the end-of-study visit to evaluate the compliance [43].

The primary outcome of ProbiotiCKD study was the urinary indican and 3-MI concentration after the treatment period.

The secondary outcomes were the after treatment concentrations of fecal *Lactobacillales* and *Bifidobacteria* and biochemistry laboratory parameters.

### Statistical analysis

The data obtained have undergone to statistical analysis conducted with SPSS software (version 20.0) and PASS 11 (NCSS LLC., Kaysville, Utah). Continuous variables were reported as either mean ± standard deviation (SD) or median and interquartile range (IQR) based on their distribution. Student’s paired *t* test or non-parametric Wilcoxon test, were used to examine the within-group differences between baseline and end-of-study visit, for normally distributed or skewed variables, respectively. Comparisons of between-groups changes from baseline were assessed by means of simple *t* test or Mann-Whitney *U* test according to distribution. Pearson’s correlation coefficient was determined for the relationship between baseline and post-treatment levels of urinary 3-MI and indican each other and with clinical, laboratory and microbiological parameters.

The level of significance was set at a *p* value < 0.05.

### Sample size calculation

Sample size was calculated considering difference in urinary indican and 3-MI concentrations after treatment as primary outcome. A sample size of 14 patients per group achieve 80% power to detect a difference of 1.1 in term of effect size (large effect size) with a significance level (alpha) of 0.05 and using a two-sided independent sample *t* test.

Sample size was calculated considering difference in urinary indican and 3-MI concentrations after treatment as primary outcome.

## Results

The anthropometric, clinical and demographic features of 28 recruited patients are resumed in Table 1. Patients had heterogeneous underlying renal disease and overall pharmacological therapy was similar in the two groups. No patient had nephrotic proteinuria or hypoalbuminemia. No therapeutic change was installed during the follow-up period. Statistical comparisons of the placebo and treatment groups were not significantly different.

**Table 1:** Anthropometric and clinical characteristics at baseline

Basal parameters	Placebo group (*n* = 14)	Probiotics group (*n* = 14)
Gender (M/F) (*n*)	6/8	9/5
Age (years)	58.2 ± 6.2	61.3 ± 5.2
Weight (kg)	74.6 ± 3.8	77.1 ± 1.9
BMI	26.2 ± 2.7	25.2 ± 3.1
Systolic blood pressure (mmHg)	130 ± 14.7	132 ± 14.2
Diastolic blood pressure (mmHg)	82 ± 6.7	84 ± 8.6
Glucose (mg/dl)	99.6 ± 14.5	98.9 ± 12.6
BUN (mg/dl)	79.5 ± 12.5	80.5 ± 10.6
Serum creatinine (mg/dl)	1.8 ± 0.3	1.78 ± 0.4
EPI-eGFR (ml/min/1.73 m^2^)	48.4 ± 7.4	49.3 ± 5.8
Cystatin C (mg/dl)	1.0 ± 0.3	0.99 ± 0.1
Uric acid (mg/dl)	4.7 ± 0.5	4.6 ± 0.4
Albumin (g/dl)	4.16 ± 0.2	4.05 ± 0.1
TSAT (%)	18.4 ± 6.8	19.4 ± 5.4
Iron (mcg/dl)	57.4 ± 9.7	60.7 ± 8.7
Transferrin (g/l)	2.25 ± 0.10	2.40 ± 0.10
Ferritin (ng/ml)	181 ± 65.5	177.9 ± 152.3
Hemoglobin (g/dl)	12.3 ± 2.6	13.2 ± 3.3
Phosphate (mg/dl)	3.2 ± 0.2	3.3 ± 0.4
Potassium (mmol/l)	4.35 ± 0.2	4.29 ± 0.1
Calcium (mg/dl)	8.8 ± 0.2	8.7 ± 0.1
Magnesium (mg/dl)	2.03 ± 0.10	2.06 ± 0.9
Sodium (mmol/l)	141.2 ± 0.8	142.1 ± 0.9
Total cholesterol (mg/dl)	165 ± 12.8	158 ± 14.3
HDL cholesterol (mg/dl)	44 ± 7	46 ± 5
Triglycerides (mg/dl)	143 ± 21	152 ± 14
iPTH (pg/ml)	203 ± 70.8	218.9 ± 56.2
C-reactive protein (mg/l)	23.6 ± 13.9	25.5 ± 15.4
Β_2_-microglobulin (mg/l)	5.6 ± 2.7	6.4 ± 1.8
Blood bicarbonate (mmol/l)	23.8 ± 2.1	24.1 ± 1.7
Estimated dietary protein intake (g/Kg/day)	0.86 ± 1.5	0.88 ± 1.3
Estimated calories intake (cal/Kg/die)	34 ± 7	33 ± 5
ACE inhibitor/AT1-receptor blockers (*n*)	12	14
Vit D analogues (*n*)	1	0
Furosemide/hydrochlorothiazide (*n*)	8	7
Oral antidiabetic drugs	4	2
Therapy with insulin	1	2
Eritropoietin	0	0
Therapy with phosphate chelants	0	0
Therapy with statins/fibrates	11	13
Kidney diseases		
Nephroangiosclerosis (*n*)	4	5
Non-nephrotic diabetic nephropathy (*n*)	5	4
Autosomal polycystic kidney disease (*n*)	2	1
Non-nephrotic unknown nephropathy (*n*)	2	2
Biopsy proven IgA nephropathy (*n*)	1	2

As for nutritionist evaluation of dietary diaries, no relevant differences were observed between groups. Moreover, baseline dietary standardized conditions were achieved (protein, potassium and fibers intake), according to protocol requirements.

Fecal *Lactobacillales* and *Bifidobacteria* concentrations were evaluated at baseline. In overall cohort, according to healthy status-associated ranges [4, 5], an insufficient fecal concentration of both *Lactobacillales* (mean 2.3 × 10^3^ CFU/gr) and *Bifidobacteria* (1.7 × 10^4^ CFU/gr) was observed in 92.8 and 95.7% of patients, respectively (Table 2).

**Table 2:** The impact of ProbiotiCKD administration protocol on fecal *Lactobacillales* and *Bifidobacteria* concentrations

Mean fecal bacteria concentration (CFU/g)	Placebo group		*p* value	Probiotics group		*p* value	*p* value placebo (AT) *vs* probiotics (AT)
	Before treatment	After treatment		Before treatment	After treatment		
*Lactobacillales*	2.3 × 10^3^	1.9 × 10^3^	ns	2.1 × 10^3^	2.2 × 10^6^	< 0.001	< 0.001
*Bifidobacteria*	1.7 × 10^4^	1.8 × 10^4^	ns	1.9 × 10^4^	2.5 × 10^7^	< 0.001	< 0.001

Data are reported as mean ± standard deviation. Statistical significance was set at a *p* value > 0.05

*CFU*/*g* colony-forming unit/g; *AT* after treatment; *ns* not significant

At baseline, a direct highly significant correlation was found between urinary indican and 3-MI each other, and of both metabolites with serum levels of C-reactive protein (CRP), ferritin and β2-microglobulin. An inverse correlation was also discovered between urinary metabolites and fecal concentrations of *Lactobacillales* and *Bifidobacteria*, as well as eGFR. No correlation with age, gender, blood pressure, and BMI was found. In Table 3 are reported all r and *p* values.

**Table 3:** Correlations between indican and 3-MI each other, and with clinical, laboratory and microbiological variables, at baseline

Parameters	Indican		3-MI	
	*r*	*p* value	*r*	*p* value
Indican	–	–	0.842	< 0.001
3-MI	0.842	< 0.001	–	–
CRP	0.891	< 0.001	0.865	< 0.001
Ferritin	0.523	< 0.001	0.529	< 0.001
eGFR	– 0.611	< 0.001	– 0.600	< 0.001
Fecal *Lactobacillales* concentrations	– 0.842	< 0.001	– 0.792	< 0.001
Fecal *Bifidobacteria* concentrations	– 0.855	< 0.001	– 0.799	< 0.001

**Fig. 2:**
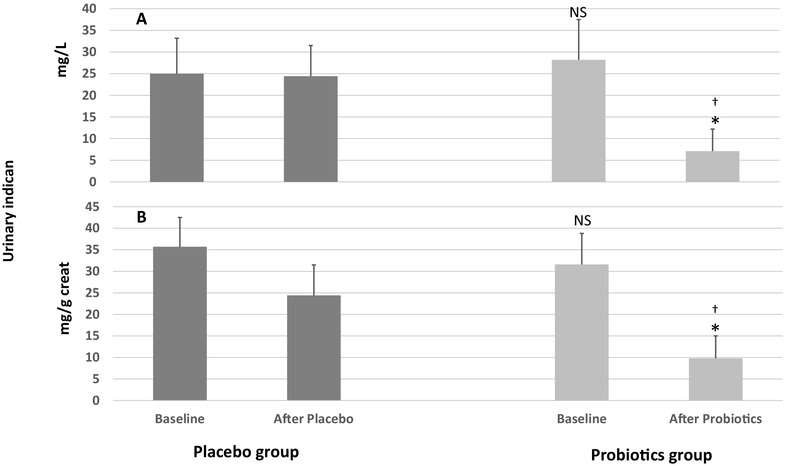
The impact of ProbiotiCKD administration protocol on urinary indican in mg/l (**a**) and and as corrected by urinary creatinine (mg/g creatininuria) (**b**). Data are reported as mean ± standard deviation. Statistical significance was set at a *p* value > 0.05. *NS* not significant vs placebo baseline; (*) vs basal (*p* < 0.001); (†) vs placebo after placebo (*p* < 0.001)

Urinary indican and 3-MI levels were used in ProbiotiCKD study to evaluate the possible predominance of either a fermentative and/or a putrefactive metabolism of gut microbiota in our CKD patients [20–23]. Remarkably, at baseline, both groups exhibited high-indican urinary levels as expression of a microbiota disequilibrium in the small bowel, thus suggesting an excessive gut bacterial fermentative metabolism. In particular, at baseline, indican averaged 28.2 ± 9.3 mg/l (31.6 ± 7.2 mg/g after correction by creatininuria) in probiotics group and 25 ± 8.2 mg/l (35.7 ± 6.8 mg/g after correction by creatininuria) in placebo group (Fig. 2).

**Fig. 3:**
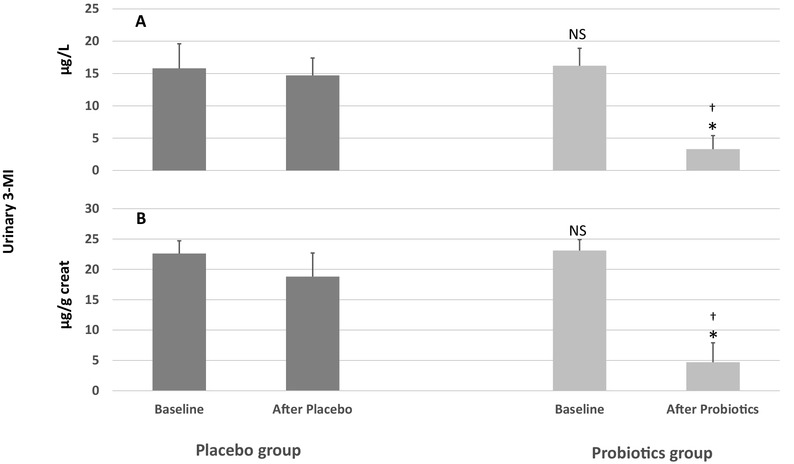
The impact of ProbiotiCKD administration protocol on urinary 3- MI in µg/l (**a**) and as corrected by urinary creatinine (µg/g creatininuria) (**b**). Data are reported as mean ± standard deviation. Statistical significance was set at a *p* value > 0.05. *NS* not significant vs placebo baseline; (*) vs basal (*p* < 0.001); (†) vs placebo after placebo (*p* < 0.001)

A putrefactive component of gut microbiota metabolism in the colon was also evident in both groups at baseline, as mean urinary 3-MI was 16.2 ± 2.7 µg/l (23.1 ± 1.8 µg/g after correction by creatininuria) in probiotics group and 15.8 ± 3.8 µg/l (22.6 ± 2.1 µg/g after correction by creatininuria) in the placebo group (Fig. 3).

Notably, gut microbiota-related variables significantly improved after treatment in the probiotics group, while no change was observed in the placebo group. In fact, mean fecal *Lactobacillales* and *Bifidobacteria* concentrations raised to 2.1 × 10^6^ CFU/g (*p* < 0.001) and 1.9 × 10^7^ CFU/g (*p* < 0.001), respectively, only in the probiotics group. The changes from baseline in fecal *Lactobacillales* and *Bifidobacteria* concentrations were significantly different between the placebo and probiotic groups (*p* < 0.001) (Table 2).

In addition, probiotics-treated patients exhibited a significant reduction of both indican and 3-MI urinary levels occurred in 96.4% (*n* = 27) and 89.2% (*n* = 24) of cases, respectively (see Figs. 2, 3 for details). By contrast, no difference was observed in the placebo group. After treatment with probiotics, in fact, mean urinary indican significantly decreased to 7.1 ± 5.2 mg/l (*p* < 0.001) [9.8 ± 4.3 mg/l after correction by creatininuria (*p* < 0.001)], while it remained significantly higher in the placebo group (24.4 ± 7.1 mg/l, *p* < 0.001), even after correction by urine creatinine (35.4 ± 4.8 mg/g, *p* < 0.001) (Fig. 2). Similarly, mean 3-MI urinary level was significantly decreased only in the probiotics group [3.3 ± 2.1 µg/l (*p* < 0.001)] as confirmed also after correction by creatininuria [4.7 ± 3.2 µg/g (*p* < 0.001)]. Conversely, no change occurred [14.7 ± 2.7 µg/l (18.8 ± 3.9 µg/g after correction by creatininuria)] in the control group after treatment. The urinary indican and 3-MI levels after the treatment period were significantly lower in the probiotics patients as compared to the placebo group (Fig. 3).

The impact of probiotics on several lab parameters is reported in Table 4. Of note, compared to baseline, mean serum iron level was significantly increased from 58.7 ± 16.7 µg/dl at baseline to 66.7 ± 14 µg/dl (*p* < 0.001) after treatment only in the probiotics group. Accordingly, mean transferrin saturation (TSAT) was also increased in response to probiotics administration from 19.4 ± 5.4% at baseline to 21.7 ± 3.7% (*p* < 0.001) after treatment. Conversely, a slight TSAT reduction was observed in the placebo group, in which mean baseline TSAT 18.4 ± 6.8% decreased to 17 ± 5.3% (*p* = 0.048). In the probiotics group, basal mean ferritin decreased from 167.9 ± 152.3 to 141 ± 113.6 ng/ml (*p* < 0.001) after the treatment cycle, while it remained stable in the placebo group.

**Table 4:** Most significant changes of lab parameters in response to ProbiotiCKD administration protocol

Lab parameters	Placebo group (*n* = 14)			Probiotics group (*n* = 14)		
	Baseline	After placebo	*p* value	Baseline	After treatment	*p* value
Iron (mcg/dl)	58.8 ± 7.6	57.5 ± 7.4	*ns*	58.7 ± 16.7	66.7 ± 14	< 0.001
TSAT (%)	18.4 ± 6.8	17 ± 5.3	0.048	19.4 ± 5.4	21.7 ± 3.7	< 0.001
Ferritin (ng/ml)	165.5 ± 115.3	167.6 ± 128.3	ns	167.9 ± 152.3	141 ± 113.6	< 0.001
C-reactive protein (mg/l)	25.8 ± 8.9	26.5 ± 3.5	ns	25.5 ± 15.4	9.7 ± 7.3	< 0.001
Total cholesterol (mg/dl)	165 ± 12.8	162 ± 13.3	ns	158 ± 14.3	151 ± 12.3	< 0.01
HDL cholesterol (mg/dl)	44 ± 7	45 ± 5	ns	46 ± 5	44 ± 4	ns
Triglycerides (mg/dl)	143 ± 21	140 ± 18	ns	152 ± 14	140 ± 12	< 0.01
iPTH (pg/ml)	203 ± 70	235 ± 67	0.03	218 ± 56	220 ± 63	ns
Calcium (mg/dl)	8.8 ± 0.2	8.7 ± 0.4	ns	8.7 ± 0.1	9.0 ± 0.3	0.03
β_2_-microglobulin (mg/l)	5.6 ± 2.7	7.5 ± 2.7	< 0.001	6.4 ± 1.8	4.1 ± 1.3	< 0.001
eGFR (ml/min/1.73 m^2^)	48.4 ± 7.4	48.6 ± 7.1	0.765	49.3 ± 5.8	49.0 ± 5.0	0.490

Data are reported as mean ± standard deviation. Statistical significance was set at a *p* value > 0.05

*ns* not significant

Moreover, mean C-reactive protein (CRP) was significantly lower after the treatment with probiotics, changing from 25.5 ± 15.4 to 9.7 ± 7.3 mg/l (*p* < 0.001). No significant variation was instead observed in the placebo group.

Conversely to placebo, iPTH did not increase in the probiotics group, in which a significant increase in mean serum calcium was also observed (see Table 4 for details).

Even total cholesterol and triglycerides both significantly decreased only in the probiotics group, as reported in Table 4. However, basal mean values of these parameters were within normal range.

Notably, mean β2-microglobulin levels showed an opposite trend in the two groups: it decreased in probiotics group from 6.4 ± 1.8 mg/l at baseline to 4.1 ± 1.3 mg/l (*p* < 0.001) after the treatment cycle and increased in the placebo group from 5.6 ± 2.7 mg/l at baseline to 7.5 ± 2.7 mg/l (*p* < 0.001) at follow-up visit. Conversely, eGFRat end-of-study visit was not different from baseline in both groups.

Compliance to treatment was overall good, as the mean number of capsules taken by patients in both groups was 438 ± 4 out of the 465 capsules provided to each patient for the entire treatment. Of note, none of the patients discontinued the treatment and no relevant side effects were reported.

## Discussion

Gut dysbiosis is defined as an imbalanced intestinal microbial community with quantitative and qualitative alterations in the composition and in metabolic activities of the gut microbiota. The criteria for dysbiosis diagnosis include evaluation of bacterial microflora and its metabolism biomarkers. Dysbiosis is a disorder often associated with diverse diseases, such as hypertension, atherosclerosis, obesity, type 2 diabetes, inflammatory bowel and cardiovascular diseases [44]. Moreover, preliminary evidences indicate that the quantitative and qualitative profile of microbiota might be altered in patients with CKD, as reported in studies performed prevalently in patients with ESRD and on chronic hemodialysis [45]. The gut dysbiosis in uremia depends on several factors. In fact, urea secretion increases in the gastrointestinal district with kidney function loss, leading to more ammonia formation that reduces commensal bacteria growth [46]. Decreased fibers dietary intake, slow colonic transit, metabolic acidosis, intestinal wall edema and possible oral iron intake might be additional pro-dysbiosis factors [47, 48]. Despite its relevance, gut dysbiosis is gaining the attention of the scientific community only in recent years and many aspects of this condition still remain open issues.

ProbiotiCKD is a single-center, prospective, randomized, placebo-controlled study designed to evaluate the intestinal microbiota and its modifications in response to a novel probiotics administration protocol in a population of CKD patients at stage 3a KDIGO. At this aim, fecal *Lactobacillales* and *Bifidobacteria* concentrations were determined and urinary indican and 3-MI levels were measured. The most surprising result observed in ProbiotiCKD is the evidence of an unhealthy gut microbiota even in patients with eGFR between 60 and 45 ml/min/1.73 m^2^. The scarce representation of fecal *Lactobacillales* and *Bifidobacteria* combined with the high-urinary indican and 3-MI levels, both widely observed in our cohort, demonstrates that even such a residual renal function is insufficient to maintain a healthy balance in gut microbiota in early CKD. Therefore, in ProbiotiCKD study, we observed a significant alteration in both gut microbiota composition and intestinal bacterial metabolism in most of the patients. In support, *Bifidobacteria* have been shown to produce short-chain fatty acids, in particular butyrate, through a cross-feeding mechanism stimulating the growth of other bacterial species such as *Lactobacillales*. Butyrate stimulates the production of antimicrobial peptides (AMp), and the expression and activity of intestinal alkaline phosphatase (IAP) with an important role in the maintenance of intestinal homeostasis [49]. We guess that the low fecal concentration of both *Bifidobacteria* and *Lactobacillales* observed at baseline, may explain the altered gut microbiota metabolism observed in our patients. In support, we found an inverse correlation between urinary indican and both *Lactobacillales* and *Bifidobacteria* fecal concentrations. The supplementation of ‘good’ bacteria strains with Ramnoselle and Bifiselle, after having improved gut ambient with Enterelle, has possibly influenced the engraftment and growth of *Bifidobacteria* and *Lactobacillales* in the gut, with reduced production of gut dysbiosis metabolites. In fact, the coexistence of abnormally high-urinary levels of both 3-MI and indican in most of patients (*n* = 26) at baseline was significantly reduced after probiotics administration with consequent correction of dysbiosis in both putrefactive and fermentative components.

It is also important to emphasize that 3-MI and indican should not be considered just as type of dysbiosis biomarkers, but also as gut-derived uremic toxins. In particular, indican causes endothelial cell dysfunction and damage and is associated with tubulo-interstitial fibrosis, aortic calcification, vascular stiffness. Moreover, in patients with renal dysfunction, indican is the predictor of CKD progression and increases the overall and cardiovascular mortality risk [50]. Similarly, as observed in human and animal studies, also 3-MI induces glomerular sclerosis, interstitial fibrosis and is a predictor of mortality and cardiovascular events in patients with CKD [51]. Therefore, dysbiosis represents a signal from gut triggering inflammation and cardiovascular damage in CKD, since the initial stages of the disease. Accordingly to Ramezani et al., not only it is time for interventions aimed at blocking microbiota-related pathogenic biochemical pathways to ameliorate uremic syndrome [52], but also to anticipate the treatment to the early stage of renal insufficiency. This strategy could contribute to reduce the high mortality and comorbidity rate observed in the advanced stages of CKD.

ProbiotiCKD is the first intervention study performed in dysbiotic patients in early CKD stages, i.e., when GFR is greater than 44 ml/min/1.73 m^2^. Actually, a previous, randomized trial tested the impact of probiotics administration on microbiota metabolites, but it was carried out in CKD patients with lower residual renal function (GFR ≤ 30 ml/min) than in our cohort. In addition, in these patients, the treatment with probiotics resulted effective in reducing p-cresil-glucuronide levels, but ineffective in lowering indican levels [53]. In comparison to our study, a more advanced stage of renal insufficiency and the different type and modality of probiotics administration might have influenced the different results.

It is evident that an optimization of probiotics administration protocols is needed. Gut dysbiosis treatment implies the use of good quality probiotics. Probiotics are live and vital microorganisms able to benefit the host if consumed in an adequate amount, as part of a food or a supplement [54]. To be considered probiotics, microorganisms have to be normal components of human gut microbiota with effective delivery in the intestinal district of at least 10^7^–10^9^ cells per day [55]. Consequently, probiotics should be resistant to sudden pH changes due to the exposure to gastric and pancreatic juice and bile. In some cases, different factors (unsuitable intestinal environment, insufficient dose, poor quality products) may interfere with probiotics effectiveness in correcting gut dysbiosis. The probiotics that we used in our study were high-quality products accomplishing to Italian Minister of Health criteria, and our novel ProbiotiCKD administration protocol was accurately projected. The rationale was based on creating a favorable intestinal environment prior to gut colonization with probiotics. Moreover, a good delivery of probiotics in the gut was assured using high doses of quality probiotics masked to the stomach acidity by a gastro-resistant film. ProbiotiCKD administration protocol revealed its effectiveness in correcting CKD-related intestinal dysbiosis. Undoubtedly, more studies comparing even other probiotics administration protocols in larger populations with comparable residual renal function, and with a longer follow-up, would be desirable.

Species of *Lactobacillus* and *Bifidobacterium* are most commonly used as probiotics, but the yeast *Saccharomyces boulardii* and some *E. faecium* are also used. A number of healthy effects are associated with usage of probiotics. In fact, their administration has been shown to stimulate the immune response, have an anti-inflammatory effect and restore gut dysbiosis [56]. *Enterococcus* species, in particular *E. faecium*, have been widely used over the last decade in the food industry as probiotics or as starter cultures because they produce bacteriocins [57]. In ProbiotiCKD protocol, a washout period with *E. faecium* was planned because *Enterococcus* species, in particular *E. faecium*, have been widely used over the last decade in the food industry as probiotics or as starter cultures to produce bacteriocins. These antimicrobial peptides are ribosomally synthesized and released in the extracellular ambient to fight competing bacterial species. Moreover, *Enterococcus* species are known to produce a range of enterocins, including enterocins A, B, I, L and P, which are active against pathogen bacteria such as *Staphylococcus aureus, Listeria* and *Clostridium* species [57]. For all these reasons, the use of *E. faecium* within our ‘sequential’ probiotics administration protocol, preliminary to gut colonization with *Bifidobacteria* and *Lactobacillales* strains, was addressed to create a favorable intestinal ambient to the engraftment of these eubiotic bacteria [58].

In our investigation, we observed intriguing effects of the tested ‘sequential’ probiotics administration on different lab parameters. First, a significant reduction of CRP levels was obtained only in the treated group. Considering that a chronic inflammation state is associated to CKD and can influence several long-term clinical outcomes (e.g., high-cardiovascular risk, anemia, and immunodepression), we believe that our observations could be relevant if translated to clinical practice. Moreover, we suggest that the anti-inflammatory effect of sequentially administered probiotics might be also considered in other patient typologies (e.g., patients with cardiac, oncologic, gastroenteric, infectious, and hepatic diseases) [59–61].

Of note, an improvement in serum iron, ferritin and TSAT limited to patients treated with probiotics was observed in our study. According to Tursi et al. [62], we hypothesize that this result might depend on both the ameliorated iron reabsorption in the gut and the reduction of inflammation. Surprisingly, no effects of probiotics were observed with respect to hemoglobin levels despite the improved iron status, but we suppose that this result could emerge in a longer observation study. We are currently running another study aimed to better characterize the interference of ProbiotiCKD protocol on specific biomarkers of both inflammation and erytropoietic activity.

As for serum lipids levels, after treatment, an improvement of total cholesterol and triglycerides was observed only in the probiotics group. However, even at baseline, lipid control was overall satisfactory in both groups, possibly due to the large employment of statins and fibrates, as reported in Table 1. Anyway, our observation confirms the results of several other studies in which the consumption of probiotics reduced the systemic cholesterol levels and caused a decrease in triglycerides as well. This result appears relevant because it suggests an additional pathway of cardiovascular risk reduction linked to probiotics use.

Focusing on another fundamental clinical feature of CKD, such as mineral bone disease (MBD), despite the short follow-up, in probiotics group, we did not observe any significant change in iPTH plasma levels, while serum calcium levels significantly improved. By contrast, iPTH increased and serum calcium remained unchanged in the placebo group. We hypothesize that the ameliorated calcium reabsorption due to gut dysbiosis correction, could have helped to prevent iPTH increase in the probiotics group. This result appears of great interest in CKD patients. A confirmation in a larger population with early stage CKD and with a longer follow-up would be an important focus for future investigations and to develop new treatment strategies aimed to prevent cardiovascular and mineral bone disease.

Another rather interesting result observed in our study is the opposite trend of β2-microglobulin in the two groups. Specifically, β2-microglobulin was correlated to urinary 3-MI and indican at baseline and decreased in the treated group after probiotics administration, while it was increased in the placebo group. Even this result is of great interest. Doubtless, further studies are needed to confirm our data and also for understanding the link existing between intestinal dysbiosis and β2-microglobulin in CKD patients.

## Conclusions

ProbiotiCKD is the first intervention study demonstrating that an intestinal mixed (fermentative and putrefactive) dysbiosis is present even in the earlier stages of CKD and that it can be effectively corrected by high-quality probiotics novel mode of administration tested in the study. Moreover, the intestinal dysbiosis correction was associated with improved CRP, iron status, iPTH and β2-microglobulin only in the treated group. Consequently, we suggest that (a) the probiotics administration protocol employed in ProbiotiCKD can represent a valid therapeutic tool for an effective intestinal dysbiosis correction; (b) probiotics administration has been associated with positive reflexes on several important lab parameters; (c) the probiotics therapy could help reduce inflammation in CKD, with possible beneficial effects on cardio-renal outcomes, particularly if the treatment is early started. The latter aspect would need a more extensive study in a larger population and with a longer follow-up.
