# Correction to: An open-label, randomized, placebo-controlled study on the effectiveness of a novel probiotics administration protocol (ProbiotiCKD) in patients with mild renal insufficiency (stage 3a of CKD)

**DOI:** 10.1007/s00394-018-1831-x

**Published:** 2018-10-09

**Authors:** Mariadelina Simeoni, Maria Lucia Citraro, Annamaria Cerantonio, Francesca Deodato, Michele Provenzano, Paola Cianfrone, Maria Capria, Silvia Corrado, Emanuela Libri, Alessandro Comi, Arturo Pujia, Ludovico Abenavoli, Michele Andreucci, Massimo Cocchi, Tiziana Montalcini, Giorgio Fuiano

**Affiliations:** 1 Nephrology Unit, Department of Surgical and Medical Science, ‘Magna Graecia’ University Hospital, Viale Europa, Germaneto Area, 88100 Catanzaro, CZ Italy; 2 Clinical Nutrition Unit, ‘Magna Graecia’ University Hospital, 88100 Catanzaro, CZ Italy; 3 Digestive Physiopathology Unit, ‘Magna Graecia’ University Hospital, 88100 Catanzaro, CZ Italy; 4 “Paolo Sotgiu” Institute for Research in Quantitative and Quantum Psychiatry and Cardiology, LUdeS, Lugano, Switzerland

## Correction to: European Journal of Nutrition 10.1007/s00394-018-1785-z

In the original publication of the article, few of the authors were missed in the author group.

The correct author group is mentioned above. The original article has been corrected.

